# A temperature-controlled chip holder with integrated electrodes for nanofluidic scattering spectroscopy on highly integrated nanofluidic systems

**DOI:** 10.1038/s41378-025-01125-9

**Published:** 2026-01-19

**Authors:** Björn Altenburger, Joachim Fritzsche, Christoph Langhammer

**Affiliations:** Department of Physics, Chalmers University of Technology, SE-412 96 Gothenburg, Sweden

**Keywords:** Nanofluidics, Nanometrology, Nanophotonics and plasmonics

## Abstract

Fluidics on the micro- and nanoscale have been revolutionary for the fields of biology and medicine, and they are gaining a strong foothold in chemistry with the rise of micro and nanoscale reactors. These systems are based on fluidic platforms crafted into polymer or silicon-based substrates, and are comprised of channels with different functions and sizes that span from the micro- to the nanoscale. However, to fully capitalize on the possibilities offered by such highly integrated fluidic systems, the periphery that connects the fluidic chip to the macroscopic world, and thereby makes it accessible for the envisioned functions and applications, is equally important but receives much less attention. Such periphery needs to be versatile and enable accurate control of pressures and flow of liquids or gases, of sample temperature, and for certain applications even electric fields. Here, we report the development of a temperature-controlled fluidic chip holder for heating and cooling that is integrated with electrodes for the creation of electric fields across the fluidic system. It interfaces 1 cm^2^ silicon-based nanofluidic chips with up to 12 fluidic connection points and optically transparent lid, that makes them compatible with optical microscopy techniques. We demonstrate the different functionalities of the sample holder by using nanofluidic scattering spectroscopy (NSS) to monitor the on-chip mixing of two different dyes, the diffusion of fluorescein into water at different temperatures, and the diffusion of fluorescein into water at different strengths of an electric field applied along a nanochannel.

## Introduction

The field of microfluidics and its evolution into nanofluidics concerns a wide range of diverse and interconnected technical applications^1–3^. At the same time, as the fluidic conditions in, e.g., blood vessels and capillaries of nearly all living organisms are in the micro- to nanofluidic range, using fluidics to artificially generate vessels for biological organs-on-a-chip is a rapidly expanding area of research^4^. Microfluidics as a scientific field emerged ca. 30 years ago^3^, when the convergence of fluid dynamics, chemistry and biology in concert with microelectronics led to an increased interest in small-scale chemical reactors^5^, lab- and organ-on-a-chip platforms^3,4^ and various handheld testing devices^6,7^. It was then the rapid development of microelectronics and the associated micro- and nanofabrication methods that enabled the step towards nanofluidics. This provided a plethora of experimental platforms to many research fields operating at the nanoscale, such as single-cell and single-biomolecule studies^1^ or heterogeneous catalysis on (single) nanoparticles^8^. Within these applications, the individual (biological or inorganic) nano-objects are often either transported, separated or forced to react with each other with the help of, for example, electrophoresis or electroosmosis^9–11^ inside a fluidic structure, such as a micro- or nanochannel^12^, requiring the possibility to apply electric fields across selected regions of the fluidic system. Furthermore, the combination of fluidics and electronics enable electric field enhancement^13^, microfluidic pumps^14^ and even field-effect transistors^15^. In the same vein, the control of temperature plays a significant role, as many of the investigated processes show a direct dependence on temperature, may it be for biological processes, such as protein dynamics^16,17^, enzymatic activity^18^ or catalysis^19^ in general. Dynamic heating and cooling of fluidic systems, possible with the here presented chip holder, can even enable valves on the nanoscale^20^. Furthermore, increasingly intricate and complex fluidic systems have been designed to include more functions and a higher level of fluidic control, requiring multiple connections between the microscopic and macroscopic world^21–23^.

**Fig. 1:**
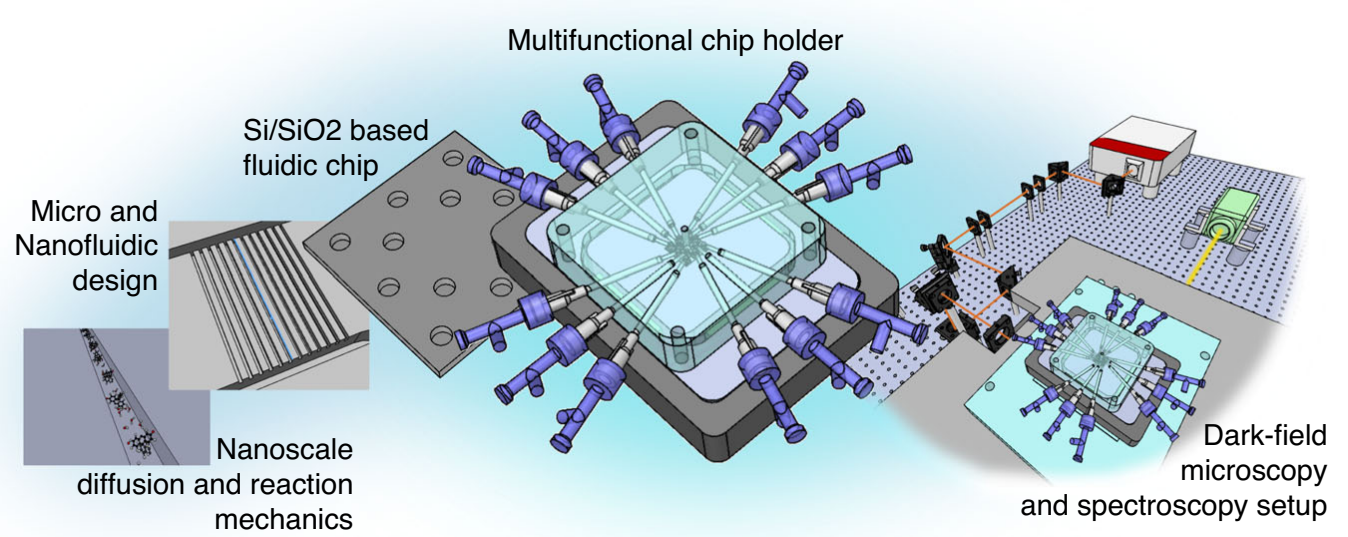
The fluidic chip holder as interface between micro- and macroscale. Research on fluids, particles, molecules and reaction mechanisms in the nanoscale regime requires a controllable fluidic environment, often in the form of a nano- and microchannel system. These systems are commonly embedded in polymer or silicon-based chips that are fabricated in clean room environments for specific experiments. To access the fluidics on those chips, a chip holder is required that ensures a facile but tight connection to macroscopic tubing and control over experimental parameters, such as temperature or electric fields. In addition, this chip holder needs to be adapted to the observation equipment, which in our case is a custom-built dark-field optical microscope

While these rapid and diverse developments in the micro- and nanofluidics arena are truly remarkable, they also make clear that this development trajectory can only continue when the evolution of fluidic chips is accompanied by a proportional maturing of their periphery, i.e., of flexible multifunctional fluidic chip holders that interface the micro-/nanofluidics with the macroscopic world (Fig. 1). Specifically, the need for ever more integrated fluidic chips with large numbers of individually addressable fluidic systems in combination with the required accurate control of environmental conditions, such as temperature, pressure and electric fields, poses a significant challenge. To this end, several approaches have been made to create fluidic connections, for example, with tubes directly attached to the often polymer-based fluidic system^24,25^ or by using pressure-based connections with O-ring-seals^24,25^ such as in this and our previous works^26–31^.

In addition to the setup-related challenges connected to the control of fluidic and experimental parameters, there is the need to be able to observe (single) molecules, nanoscale objects or chemical/biological processes occurring inside the fluidic system in situ. Such observations are most commonly done with brightfield^32,33^ or darkfield optical microscopy^34,35^, or with X-ray techniques^36^ and in-situ transmission electron microcopy (TEM)^37,38^ as two emerging highly promising alternatives and/or complements to optical methods.

As a final key aspect, we highlight the importance of both scalability and reduction of the environmental footprint of the micro- and nanofabrication of fluidic devices or “chips”, in combination with the aforementioned increasing importance of highly integrated fluidic designs. Technically, this is best achieved by miniaturization of the fluidic chips since in this way a larger number of devices is produced per silicon wafer and, e.g., per emission of highly climate-potent perfluorocarbon species widely used for reactive-ion etching. While certainly feasible from the micro- and nanofabrication perspective, the miniaturization of fluidic chips poses a challenge in terms of their handling and flexible interfacing with a macroscopic chip holder that connects to the fluidic systems integrated with the chip, as well as provides additional functions, such as temperature and pressure control, electric contacts, and a suitable interface to desired readout techniques.

To address this challenge, in this work, we present a temperature-controlled nanofluidic chip holder (Fig. 1) with integrated electrodes that is compatible with optical microscopy readout and that can host a 1 × 1 cm^2^ sized silicon/silicon dioxide fluidic chip with up to 12 independent in- or outlets that connect to independent fluidic systems. Importantly, as a consequence of their small size, 52 of these chips can be produced per 4 inch wafer. We demonstrate the different functionalities of the chip holder on the basis of our recently introduced nanofluidic scattering spectroscopy readout method (NSS)^28^ by investigating the on-chip mixing of Fluorescein and Brilliant Blue solutions, and the dependence of Fluorescein diffusion into water on both temperature and an applied electric field inside individual nanofluidic channels.

## Results and discussion

### A temperature-controlled chip holder with integrated electrodes

**Fig. 2:**
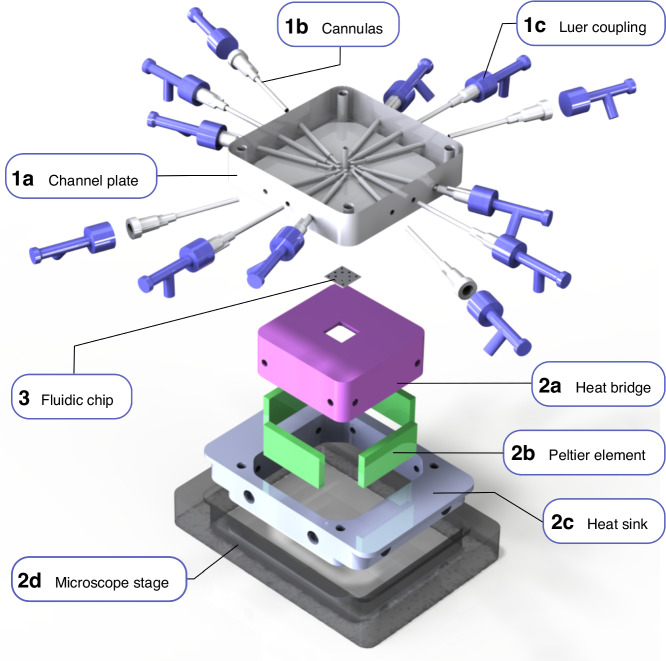
Overview of the chip holder assembly. The channel plate (1a) that sits atop the fluidic chip is the most central part of the chip holder since it interfaces the macroscopic liquid inlets, electrodes and temperature probes with the microfluidic system on the silicon-based fluidic chip (3). It is made from optically transparent acrylic glass to enable visual observation of the 12 in- and outlet reservoirs during an experiment using optical microscopy. It houses the cannulas (1b) that enable the connection between Luer-Lock couplings (1c) and the inlet reservoirs. The channel plate is pressed against the fluidic chip (3) and O-rings seal the connection to the 12 reservoirs on the chip. The chip itself rests in the heat bridge (2a) that provides mechanical support but also connects the chip thermally to four Peltier elements (2b). Depending on the direction of the electric current applied, these elements transport heat to or from the heat bridge and thus heat or cool the fluidic chip. Since the heat bridge only contacts the Peltier elements, the air gap between the heat bridge and the heat sink (2c) inhibits thermal exchange between the two. Furthermore, the heat sink mechanically connects the chip holder to the sample frame that sits on the inverted microscope stage (2d)

The fluidic setup we introduce has two major constituents: the temperature-controlled fluidic chip holder with integrated electrodes (1a–2c in Fig. 2) and the Si/SiO_2_-based fluidic chip (3 in Fig. 2). To maximize versatility, user-friendliness and scalability of the fabrication process, the chip is quadratic with a side length of only 10 mm to introduce symmetry that enables it to be placed in the chip holder at rotations of 90°. Furthermore, the chosen size and format are large enough to enable complex micro and nanofluidic systems and easy handing, while also being small enough to be produced in large quantities per wafer. The key limiting factor in this respect is the number of connection points between the fluidic systems on the chip and the holder that are practically feasible in terms of, e.g., the dimensions of the O-rings used for sealing and their ability to supply and withstand different pressurized liquids and gases from a chemical perspective. Furthermore, the insertion of electrodes in these connection points enables the application of electric fields. For a 1 cm^2^ chip, this results in a design with 12 fluidic connection points per chip, which are contacted with the tailored channel plate of the holder (1a in Fig. 2). The channel plate features inserted cannulas (1b in Fig. 2) onto which Luer-Lock couplings (1c in Fig. 2) are mounted, through which easily exchangeable syringe needles for liquid insertion or electrodes can be inserted to contact the liquid reservoirs of the 12 connection points of the chip.

The fluidic chip itself is placed in a quadratic hole with stepped edges that is situated on top of an aluminum cup that also serves as heat bridge (2a in Fig. 2). This part not only provides mechanical support for the chip and space for the microscope objective of an inverted microscope but also serves as thermal connection between the chip and four Peltier elements (2b in Fig. 2) that are pressed against its sides and the outer frame (2c in Fig. 2). They enable both heating and cooling of the chip. The outer frame serves as mechanical support for the whole chip holder assembly and functions as heat sink for the Peltier elements. This heat sink function is further enhanced by the tight mechanical connection to the microscope stage (2d in Fig. 2). Below, we will discuss the components of the chip holder, as well as the design and functionality of the nanofluidic chip used in more detail.

#### Channel plate

**Fig. 3:**
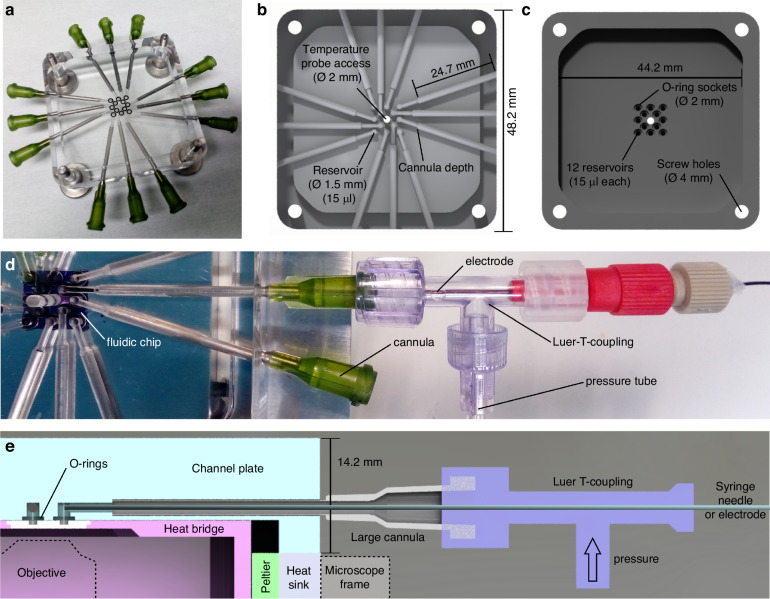
Channel plate and its functions. **a** Photo of the channel plate that features 12 macroscopic in/outlet channels that can connect to a fluidic chip with up to 12 inlet reservoirs. It is made of transparent acrylic glass that enables visual inspection and monitoring of the inlet reservoirs. The cannulas are glued into the outer part of the channels that are machined into the channel plate and provide the connection for Luer couplings. **b** Render image of the channel plate when viewed from above with relevant dimensions indicated. The hole in the middle provides access to the fluidic chip surface for a temperature probe or enables optical microscopy in transmission mode. **c** Render image of the backside of the channel plate. The liquid reservoirs of the channel plate are connected to the fluidic chip via O-rings, which are socketed into circular outcroppings. Each reservoir has a volume of 15 microliters (µl). **d** Photo of the Luer-Lock coupling at the end of the inserted cannula. The use of lockable connectors is important since the chip holder must handle high pressures (up to 4 bar) that are applied to establish liquid flow through the nanofluidic systems on the fluidic chip. If not in use, the couplings can be capped or left open for pressure release. In the image, a Luer-T-coupling is connected to the cannula to enable insertion of an electrode, while at the same time also enabling pressurization of the reservoir. **e** Schematic cross section through the chip holder along one channel with all key features and functions indicated. Below the fluidic chip, the position of the microscope objective is also shown

The channel plate is machined from acrylic glass for optical transparency (Fig. 3a) and connects the macrofluidic system of the chip holder with the micro- and nanofluidic systems of the chip. Its design is governed by the following technical factors: (i) size of commercially available O-rings and their ease of handling; (ii) the dimensions and lengths of commercially available syringe needles used for the insertion of liquids; (iii) the size of the fluidic chip, which should be as small as possible from a fabrication yield per wafer perspective; (iv) the number of connection points used as inlets that can fit onto the chip and can be contacted via the channel plate in a reasonable way. Based on these boundary conditions, we designed the channel plate as depicted in Fig. 3a–c, where 13 circular connection positions with 2 mm diameter are arranged in a square packing within the 1 cm^2^ of the fluidic chip. The number of connections is the result of an optimization that considered the available chip surface, the size of commercially available O-rings, the manufacturing techniques used for the channel plate and the goal of being as efficient as possible considering chip fabrication and material. In our earlier works^26,30,31^ we used fluidic systems that required 4 inlets and were able to fabricate 16 of these systems per 4-inch silicon wafer, whereas now we can fabricate 156 of such systems per wafer. In addition, with an increased number of possible connections to the fluidic chip, more advanced fluidic systems can be implemented, as demonstrated in another of our earlier works^28^, where we were able to introduce a separate reference system thanks to 12 connections points per fluid system.

Based on these earlier experiences, 12 of the 13 possible connection points in the fluidic chip design presented here can be reached by horizontal channels in the channel plate and are sealed with O-rings with an inner diameter of 1.5 mm, whereas the position in the center is used for direct access to the fluidic chip. While primarily designed to host a temperature probe, this central hole in the channel plate could also be used for, e.g., optical microscopy in transmission mode through optically transparent fluidic chips^32–34^ or for X-ray-based readouts^36^. The 12 fluidic connection points of the chip each have a vertical cylindrical cavity above them (1.5 mm in diameter, 15 µl volume), integrated in the channel plate, with a socket to hold the O-ring in place (Fig. 3c). The horizontal channels in the channel plate that lead to these cavities are machined at a specific in-plane angle that is defined by the size of the Luer-lock couplings at the end of each inserted cannula (Fig. 3a, b). The couplings need to be far enough apart to allow unhindered operation, while at the same time being relatively close, such that the channels are as short as possible and about the same length for each of the 12 positions. The overall dimension of the channel plate is defined by the frame of the microscope stage used, which in our case is a Mad City Labs RM21 (3 in Fig. 2), and the size of the heat bridge, which is located under the channel plate inside a cavity. Consequently, the quadratic channel plate has an outer side length of 48.2 mm (Fig. 3b) and a cavity with 44.2 mm side length machined into its back side (Fig. 3c). The four holes in the corners of the channel plate are used to mechanically connect it to the outer frame and establish a tight connection to the fluidic chip by pressurizing the O-rings. Finally, we note that we have chosen acrylic glass for the channel plate due to its optical transparency, facile machinability and low thermal conductivity. However, we highlight that acrylic glass has a low resistance to organic solvents and a relatively low glass transition temperature of 115 °C^39^. This limits its applicability in terms of chemicals used and temperature range. However, thanks to the modular design of the entire chip holder, this limitation can easily be amended by machining a channel plate from a different material, if required.

Focusing next on the external connections to the channel plate, the Luer-Lock connections mounted on the glued cannulas can be used for multiple purposes. In the example depicted in Fig. 3d, a Luer T-connector is first connected to the cannula, such that a syringe needle or electrode can be inserted, while at the same time being able to apply pressure to the same connection point through the side port of the T-connector that is connected to a pressurized gas inlet (e.g., N_2_). A schematic cross-section through the chip holder along one horizontal channel is depicted in Fig. 3e. It shows how an electrode (or syringe needle for liquid insertion) is inserted through the T-coupling and the cannula to reach directly above the connection points on the fluidic chip. In this way, each of the 12 connection points on the chip can be functionalized individually with different liquids, gases, electrodes and pressures.

#### Heat transport system

**Fig. 4:**
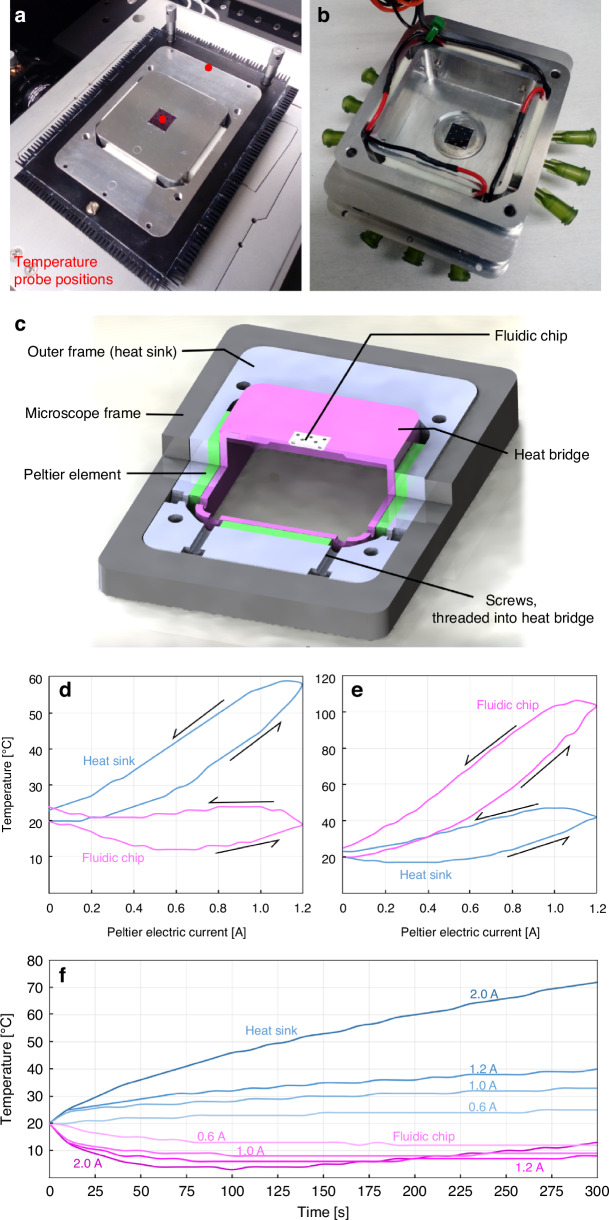
Heat transport system. **a** Photograph of the chip holder with the channel plate removed, revealing the fluidic chip resting in the heat bridge. The Peltier elements are visible as the white ceramic parts between the heat bridge and the outer frame, on which heat dispersing elements are attached. The placements of the temperature probes for **d**–**f** are indicated with red dots. **b** Backside of the chip holder, showing the connecting wiring and the cavity of the heat bridge where the microscope objective is located when the holder is mounted on the inverted microscope. **c** Render image with cut-out showing the components involved in heat transfer. The Peltier-elements function as heat pumps in either direction, depending on the direction of the electric current, and use the outer frame and the table of the microscope as heat reservoir. Since they are the main mechanical connection between the heat bridge and the heat reservoir, the chip can be cooled or heated effectively. **d** Measured temperature at the position of the fluidic chip (heat bridge) and of the heat sink as function of Peltier electric current when operating the system in cooling mode. We find 0.6 A as the optimal current to enable a steady cooling down to 12 °C. **e** Measured temperature at the position of the fluidic chip (heat bridge) and of the heat sink as function of Peltier electric current when operating the system in heating mode. Since in this mode the ohmic heating works in favor of the temperature change of the chip, a higher temperature difference can be reached with the same current compared to cooling mode operation. **f** Temperature of the chip and the heat sink over time when cooling at a fixed Peltier current. It is evident that a higher current causes faster cooling and to lower temperature but only transiently since the whole system starts to heat up eventually due to the excess heat generated by the Peltier element. Lower currents provide a more stable temperature over time, but cannot reach temperatures below 10 °C

Being able to control the temperature of the fluidic system is critical for many applications, e.g., when studying protein interactions^40^ or when investigating the catalytic performance of single nanoparticles^41,42^. In the chip holder introduced here, the control of the fluidic chip temperature is managed by the heat bridge, the heat sink (outer frame), and the four Peltier elements. The use of Peltier elements is motivated by three main aspects: (i) Their working principle is based on electronic transport phenomena and does therefore avoid any mechanical vibrations, increasing the stability of the setup and decreasing the noise level of measurements. (ii) Their bidirectional heat transport properties, allowing heating and cooling of the fluidic chip dependent on the direction of the electric current. Peltier elements are with that one of the few temperature control devices used in microfluidics that can actively control the temperature of the system in both ways^43^. (iii) Peltier elements are driven and controlled by electric current, allowing direct and fast control of chip temperature and facile integration into automated experimental systems. In addition, use of Peltier elements that are placed outside of the fluidic chip (cf. Josephson et al.^18^) reduces fabrication complexity (in contrast to previous designs where we used on-chip heaters^27^) and frees up space that can be used for other features, e.g., an increased number of connection points in our case. Figure 4a shows the chip holder installed on the inverted microscope stage with the channel plate removed. Viewed from below, the wiring of the four Peltier elements that connects them in series is revealed (Fig. 4b).

As evident from the schematic cross section of the entire device, the fluidic chip sits in the center of the heat bridge, which in turn is separated from the heat sink by the four Peltier elements (Fig. 4c). The heat sink (outer frame) is fitted precisely into the frame of the microscope stage. In addition, heat dispersing elements are attached to the microscope frame to facilitate efficient heat dissipation. Notably, the heat bridge is only in direct contact with the Peltier elements, the O-rings to the reservoirs on chip, and the screws on the side that hold the heat bridge in place. In combination with this design, the small air gap between channel plate and heat bridge enables good thermal insulation, which is crucial especially when the chip is to be cooled. Good thermal contact between heat bridge and Peltier elements is established by heat conductive paste (see “Methods”).

To characterize the heating and cooling properties of our system, we recorded the temperature at the center of the fluidic chip and at the edge of the heat sink, as indicated by the red dots in Fig. 4a, as function of applied electric current to the Peltier elements for cooling (Fig. 4d) and heating (Fig. 4e), respectively. The working principle of a Peltier element is to apply a voltage across a p-n junction, in which the flowing current removes heat from one side of the device (the cooling side) and transports it to the other one (the heating side), thereby creating a temperature difference between its two sides^44^. Hence, when chip and heat bridge are to be cooled, the Peltier elements transport heat into the heat sink. While a high electric current enhances this effect, it will also increase the heat that is generated by the ohmic resistance of the Peltier device, which leads to an optimal current for maximum cooling effect. Since this point of optimal cooling depends on how effectively the heat sink can disperse the excess heat and on what temperature difference the Peltier elements of the holder can maintain, to characterize the thermal properties of our holder, we mapped both its cooling and heating response by systematically in-/decreasing the applied current.

Starting with operation in cooling mode, we increased the Peltier current in 0.05 A steps with 2 min dwell-time per step up to 1.2 A and back to 0 A, while measuring the chip and heat sink temperatures (Fig. 4d). During the increase of the current, a minimum chip temperature of 12 °C is reached at 0.6 A, while the heat sink temperature reached 30 °C. A further increase in Peltier current then increases the heat sink temperature up to 57 °C at 1.2 A. As evident, at these high currents, the heat bridge, and thus the fluidic chip, cannot be cooled effectively anymore due to the significant ohmic losses, and their temperature therefore starts to rise. Upon subsequently decreasing the Peltier current, we see first a short transient increase in heat sink temperature due to a delayed response of the system, before the temperature drops back to room temperature (Fig. 4d). However, this curve does not follow the same path as when the electric current was increased, i.e., it exhibits hysteresis, since the passive cooling of the heat sink is slow. The same hysteretic behavior is observed for the chip (Fig. 4d).

To analyze the thermal response of the system in heating mode, we executed the same procedure but with inverted Peltier current (Fig. 4e). In this case, the ohmic heating works in favor of the overall heating process, which means that heating is significantly more efficient than cooling. This is manifested in a chip temperature of 112 °C for an applied Peltier current of 1.2 A, while the heat sink remains at 42 °C. A similar hysteresis as during cooling is also observed, for the same reason.

As the final analysis, we monitored the temperature response of the system in cooling mode over time when specific Peltier currents are applied (Fig. 4f). Evidently, the temperature of the heat bridge and chip can transiently be as low as 4 °C when a current of 2 A is applied. However, this relatively high electric current deposits a large amount of excess heat in the whole system through the ohmic resistance of the Peltier elements, such that after 100 s, the temperature of the fluidic chip starts to rise rapidly, as the temperature of the heat sink steadily increases up to 72 °C after 300 s. A decrease in Peltier current mitigates this problem and at 0.6 A the lowest temperature of 12 °C is steadily maintained.

### Nanofluidic chip and nanofluidic scattering spectroscopy readout

While the chip holder defines the overall size of the fluidic chip to 1 cm^2^ and the positions of the 12 connection points, the layout of the micro- and nanofluidics on the chip can be designed freely and thus tailored to a specific application, such as for example droplet generation^45^ or single particle catalysis batch reactors^46^. Here, we use a fluidic system design that we have introduced earlier for nanofluidic scattering microcopy^29^ (NSM) and spectroscopy^28^ (NSS).

**Fig. 5:**
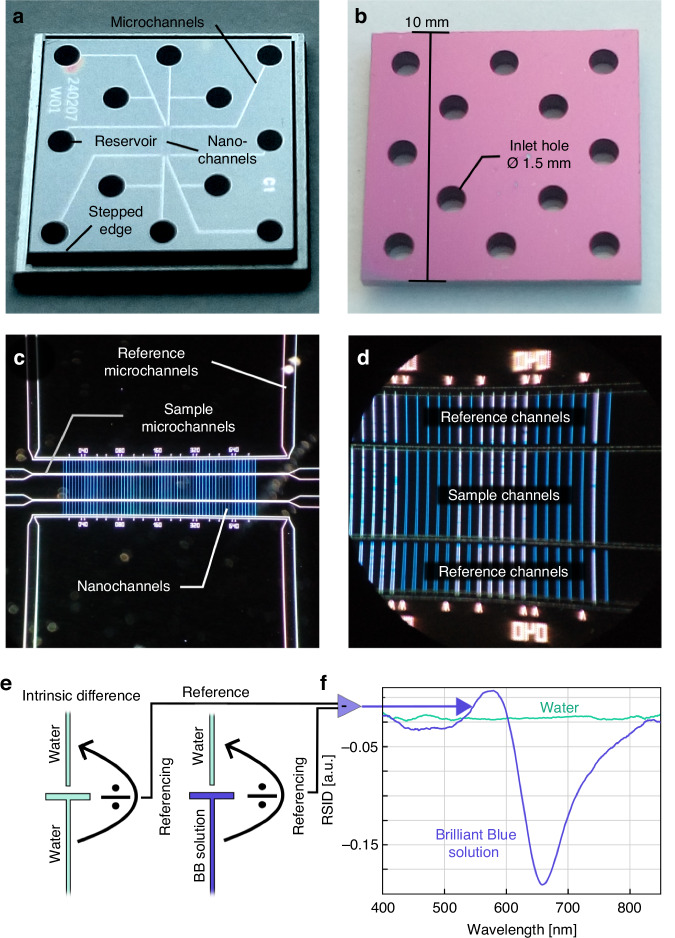
Layout of the micro- and nanofluidic systems on the fluidic chip and nanofluidic scattering spectroscopy readout. **a** Photograph of the glass-lid side of the fluidic chip with 12 in/outlets that enable 6 independent fluidic systems. The glass lid is 175 µm thick and optically transparent, and thus makes it accessible to optical microscopy and spectroscopy. The micro- and nanofluidic systems are fabricated into the 250 nm thick thermal oxide of a silicon wafer onto which the glass lid is thermally bonded. We highlight the step at the edges of the chip that enables its clamping to the heat bridge, while at the same time being coplanar with its backside. **b** Photograph of the inlet (silicon) side of the fluidic chip showing the 12 inlet holes that have been etched through the chip. **c** Dark-field microscopy image of the center of the fluidic system. The non-diffraction-limited microchannels (50 µm wide, 2 µm deep) are visible by the light scattered from their side walls, whereas the smaller nanochannels appear as single vertical lines since their scattering image is diffraction limited. In total, the array of parallel sample and reference nanochannel pairs features 31 channels with 200 nm width and 200 nm depth, and 31 channels with 100 nm width and 200 nm depth. For the experiments below, we used one of the channels in this array. **d** Section of the nanochannel system imaged at higher magnification. In this design, the sample nanochannels in the center connect to 5 µm wide and 2 µm deep microchannels on their in- and outlet sides. Each sample channel is accompanied by two colinear reference channels used for optical referencing in NSS^28^. They are also connected to their independent microfluidic system. This enables independent fluidic operations in the sample and reference fluidic systems. **e** Schematics of the NSS spectra acquisition and referencing scheme^28^ discussed in detail in the main text. First, the intrinsic difference between sample and reference nanochannels is determined before the solution in the sample is exchanged. As a result, the relative scattering intensity difference (RSID) is recorded as function of wavelength, resulting in so-called RSID spectra^28^. **f** RSID spectra obtained for a sample nanochannel filled with water and a 25 mM Brilliant Blue (BB) dye solution, respectively. The RSID-spectrum for water is a flat line, as expected when also the reference channel is filled with water. The Brilliant Blue dye spectrum exhibits the characteristic RSID peaks corresponding to the absorption bands of the dye^28^

In general, our fluidic chips are micro- and nanofabricated using a 1 mm thick silicon wafer with a 250 nm thick thermal oxide layer as the substrate (Fig. 5a, b, see Methods for details). After fabrication, the fluidic systems are hermetically sealed by bonding a 175 µm thick Borofloat 33 wafer onto the oxide into which the fluidics have been crafted. This makes the 12 connection points, which are etched through the Si wafer, the only access to the fluidic system(s). The chip also has a stepped edge, such that it can rest securely in the heat bridge without protruding from its surface. Figure 5c shows a darkfield optical micrograph of the core region of the fluidic system where microchannels that connect to the 12 connection points also connect to arrays of parallel nanochannels.

Focusing on the nanofluidic system on the chip, we first note that it is optimized for NSS optical readout and thus features the same design as in our earlier work^28^. Specifically, it is comprised of two sets of optical reference nanochannels (200 nm deep and 200 nm, respectively 100 nm, wide) above and below a central set of sample nanochannels with the same respective dimensions (Fig. 5d). In this design, the sample channels are used for the actual experiments and are connect to an in- and outlet microfluidic system on either side that enables efficient establishing of liquid flow through these nanochannels. The reference channels on the other hand are arranged colinearly to the sample channels and only on one side connected to a microchannel that, in turn, has no connection to the fluidic system of the sample channels. In this way, the reference nanochannels can be filled independently with the solvent used in the experiment at all times, and thus be used for optical referencing in an NSS experiment in the same way as in a dual-beam spectrophotometer^28^.

Conceptually, in NSS the light scattering intensity from a single nanofluidic channel is recorded as function of wavelength and treated according to the procedure depicted in Fig. 5e and reported earlier^28^. It can be summarized in the following subsequent steps:

(i) We fill both the reference and sample channel systems with the solvent of choice (here water) and place the sample/reference nanochannel pair in the slit of the spectrometer. Subsequently, a scattering spectrum (or several from different positions – depending on the specific experiment) is recorded from the reference channel and the sample channel. To record these spectra, we bin the signal from 21 pixels along the respective nanochannel, which corresponds to a ca. 15 µm long fraction of the entire channel, to reduce noise. Subsequently, we divide the obtained water-filled sample channel spectrum by the water-filled reference channel spectrum to obtain an “*intrinsic difference spectrum*” between sample and reference channel.
(ii) We exchange the water in the sample channel system by an aqueous solution of the compound of interest (here as first example a Brilliant Blue dye), by exchanging the liquid in the corresponding reservoir and establishing a flow through the nanochannel by applying pressure on the reservoir. The measured scattering spectrum from the sample channel is then divided by the simultaneously obtained spectrum of the water filled reference channel.
(iii) As a final step, we subtract the intrinsic difference spectrum measured in step (i) from the referenced sample spectrum obtained in step (ii), to account for the intrinsic differences in scattering profile that (may) exist between a reference and sample nanochannel when they are filled with the same liquid, e.g., due to slightly different dimensions or surface roughness. This results in what we call the *“relative scattering intensity difference”* (RSID) spectrum, as exemplified on a 25 mM Brilliant Blue aqueous solution flushed through the sample nanochannel (Fig. 5f). The RSID contains spectral features characteristic for the solute(s) in the nanochannel and can be back-calculated to an extinction coefficient spectrum using a formalism we have introduced earlier^28^.

### NSS monitoring of on-chip mixing and exchange of dye solutions

**Fig. 6:**
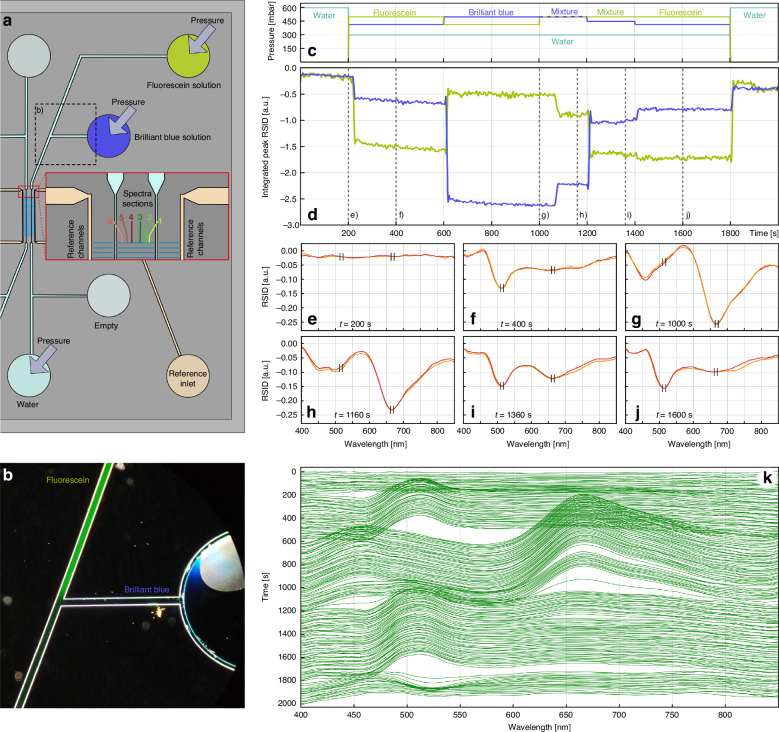
Dye exchange and mixing monitored with nanofluidic scattering spectroscopy (NSS). **a** Schematic of one side of the fluidic system on the 12-inlet chip where one inlet to the sample nanochannel system is filled with 50 mM Fluorescein solution, a second inlet with a 50 mM Brilliant Blue dye solution, and a third inlet with water. The reference fluidic system is filled with water. The empty reservoir will be used as liquid outlet during the later experiments depicted in Figs. 7 & 9. The inset shows a zoomed-in section at the start of the nanochannel array, where six different sections of a specific sample nanochannel are indicated and color-coded. Each section thus corresponds to a 15 µm long fraction of the in total 120 µm long sample nanochannel. Hence, each section corresponds to 21 binned pixels on the CCD-camera image used for NSS readout from each of the six sections and a sample volume of only 600 attoliter. **b** Dark field microscopy image of the dashed region marked in (**a**) taken while applying 500 mbar pressure to the Brilliant Blue and Fluorescein reservoirs to enable their mixing at the intersection point of the two microchannels that exit the respective reservoirs. **c** Pressures at the respective inlets during the dye exchange and mixing experiment. **d** Time traces of the integrated RSID amplitude measured at 520 $\pm$ 5 nm for Fluorescein (see **f**, **j**, also Fig. S1b, d for corresponding RISD and molar extinction coefficient spectra) and at 670 $\pm$ 5 nm for Brilliant Blue (see **g**, Fig. S1a, c). This reveals the presence and change in concentration of the respective species in the sample nanochannel. **e**–**j** Selected RSID spectra for nanochannel sections 3 and 4 along the experiment timeline that reveal the presence and transient mixing of the dyes at *t* = 1360 s. The integration intervals used for the time trace in (**d**) are indicated in black. **k** Ridge plot of the—for clarity inverted—RSID spectra over time revealing the spectral signatures of the two dyes. The transition between them can be seen as a distinct transition from the strong peak at 670 nm (Brilliant Blue) to the lower peak at 520 nm (Fluorescein). We note that while the molar concentrations of the dyes are equal, their molar extinction coefficients are different^28^, which is the reason for the stronger RSID peak of Brilliant Blue

As a first example to illustrate the function of the chip holder, we demonstrate how the 12 inlets of the chip can be used to dynamically exchange and mix liquids on-chip, and how this can be analyzed using NSS. For this purpose, we filled the reference channel systems with water, as well as the fluidic system on one side of the sample nanochannels. On the other side of the sample nanochannel array, we use our chip design that features a T-connection of two microchannels, each with its own inlet reservoir that we filled with a 50 mM aqueous Brilliant Blue and 50 mM aqueous Fluorescein solution, respectively (Fig. 6a). On the other side of the nanofluidic system, we filled one reservoir with pure water and left one empty as storage for used solution. With this arrangement, by balancing the pressures applied to these three reservoirs (Fluorescein, Brilliant Blue, water), we can control the solution that flows through the nanochannels without manipulating the chip holder itself, which is critical for in situ NSS readout. Specifically, by simultaneously applying 500 mbar to the two dye-filled reservoirs, we can mix the two solutions at the intersection of the microchannels exiting the respective reservoirs (Fig. 6b).

To demonstrate and evaluate the on-chip fluid mixing dynamically, we applied the pressure scheme depicted in Fig. 6c to the three reservoirs filled with (i) water, (ii) 50 mM Brilliant Blue solution and (iii) 50 mM Fluorescein solution. To evaluate the liquid exchange process, we focus on a single nanochannel with 200 nm × 200 nm cross section and divide its NSS readout into 15 µm long sections by binning 21 pixels on the CCD-camera image for each section (each corresponding to a volume of 600 attoliter) from which we extract independent RSID spectra. We then trace the amplitude of the main RSID peak^28,46^ of Brilliant Blue (670 nm) and of Fluorescein (520 nm) over time by integrating the RSID amplitude from 665 to 675 nm and from 515 to 525 nm, respectively (indicated in Fig. 6e–j, see also Fig. S1 for a correlation between absorbance and RSID spectra). The corresponding result shown in Fig. 6d for nanochannel section 3 (the results are similar for all other sections and thus not shown) is a set of steps over time, where a larger negative RSID amplitude signifies the presence of a higher concentration of the respective solute. When the first exchange from water (flat spectrum, Fig. 6e) to Fluorescein is triggered at 200 s, we applied 500 mbar pressure to the Fluorescein reservoir, 416 mbar to the reservoir of Brilliant Blue and 300 mbar to the water reservoir, to avoid it being contaminated by Fluorescein. The corresponding integrated RSID response from the nanochannel taken at a wavelength of 520 nm is delayed by 15 s, which corresponds to the time the solution needs to flow from the reservoir to the nanochannels. Notably, the RSID signal taken at 670 nm changes slightly as well, since the RSID response of Fluorescein is non-zero also in this regime (Fig. 6f).

At 600 s the pressures are changed to enable the inflow of Brilliant Blue. The corresponding delay in RSID response is shorter due to the shorter distance between the Brilliant Blue reservoir and the nanochannel in focus. Furthermore, the integrated RSID amplitude is larger compared to the same concentration of Fluorescein due to the different molar extinction coefficients of these dyes^28^. At this point, the RSID spectrum from the nanochannel changes significantly, i.e., the peak at 520 nm (Fluorescein) has vanished and a new, stronger peak at 670 nm (Brilliant Blue) has emerged (Fig. 6g).

In the next step at 1000 s, we increased the pressure on both dye reservoirs to 500 mbar, such that mixing of the two dyes occurs in the microchannels, as shown in Fig. 6b. As a result, the concentration of Brilliant Blue drops, as manifested in the decrease of the RSID amplitude at 670 nm, while there is an increase in the amplitude of the Fluorescein peak at 520 nm that signals the presence of Fluorescein in the nanochannel, as corroborated by the presence of the RSID peaks of both dyes in the spectrum (Fig. 6h). As expected, by decreasing the pressure applied to the Brilliant Blue reservoir by 50 mbar at 1200 s, the dye mixture ratio can be changed, as revealed both in the integrated RSID time trace (Fig. 6d) and the corresponding RSID spectra (Fig. 6i). As the last step, at 1400 s, we reestablished the same pressure condition as at 200 s, indeed restoring the corresponding integrated RSID peak response and RSID spectrum (Fig. 6j). The time evolution of the spectral RSID response of the entire experiment is depicted in the ridge plot in Fig. 6k, where we have inverted the RSID amplitude for clarity. In summary, this experiment demonstrates the facile switching between different solutions and the creation of their mixtures on-chip, as well as the in situ readout of these fluidic operations using NSS, enabled by our chip holder and the 12-inlet fluidic chip design.

### Temperature-dependent Fluorescein diffusion

**Fig. 7:**
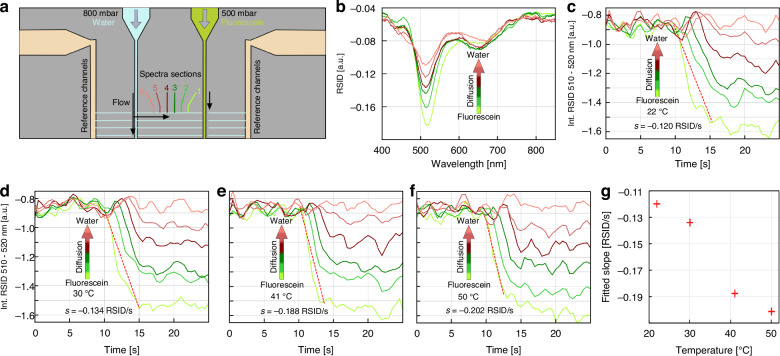
Diffusion of Fluorescein into water at different temperatures. **a** To prepare for this experiment, a pressure of 500 mbar was applied to a reservoir filled with 50 mM Fluorescein solution which is connected to one side of the sample nanochannels via a microchannel. At the same time, 800 mbar were applied to a water-filled reservoir on the other side of the sample nanochannel system to establish a water flow through the nanochannels into the Fluorescein-filled microfluidic system. At *t* = 10 s, all pressures were released, leading to Fluorescein freely diffusing into the water-filled nanochannels, whereof one channel (200 × 200 nm^2^ cross-section) was monitored by NSS, divided into six sections indicated and color-coded. **b** RSID spectra of the six nanochannel sections for 50 °C and at *t* = 20 s. **c**–**f** Time traces of the RSID intensity integrated from 510 to 520 nm (Fluorescein peak) are measured at 22 °C (**b**), 30 °C (**c**), 41 °C (**d**), 50 °C (**e**) for the six color-coded nanochannel sections upon diffusion of the 50 mM Fluorescein solution into water in the channel. Nanochannel section 1 is closest to the Fluorescein- filled microchannel and thus exhibits the strongest response at all temperatures, whereas the RSID-amplitude from channel section 6 closest to the water-filled microchannel stays constant during the whole experiment. Defining the rate of RSID change as a descriptor for the diffusion rate at the four different temperatures, it is evident that steady state is reached faster at the higher temperatures, and that the slope during the transition (indicated with a red dashed line) becomes steeper. **g** Comparison of the slope values (RSID change per unit time) for the different temperatures, showing that a higher temperature leads to faster diffusion, as expected. The red crosses mark the value of the slope of the red dashed lines fitted to the RSID time traces shown in (**c**–**f**)

As the next example to showcase the functionality of our chip holder, we combine the fluidic control of the dye solutions with the possibility to vary the temperature of the same fluidic chip used above, as well as with the spatially resolved NSS readout using the six channel sections. Specifically, we set up a diffusion experiment, where a 50 mM aqueous Fluorescein solution is let to diffuse freely into a water-filled nanochannel monitored in situ using NSS. As the starting point, one reservoir on one side of the array of nanofluidic channels is filled with the Fluorescein solution, while one reservoir on the other side of the nanochannels is filled with pure water (Fig. 7a). The reference nanochannels are also filled with water, while all other reservoirs on the 12-inlet chip are filled with water as well, but never pressurized. By, as the starting condition, applying a pressure of 800 mbar to the water reservoir and 500 mbar to the Fluorescein reservoir, the respective connecting microchannels are flushed with their respective solutions, while the nanochannels are flushed with water due to the higher pressure applied to the water reservoir. To initialize the free diffusion of Fluorescein into the nanochannel, we released all applied pressures from the fluidic system to terminate all convective flow and monitored over time the response of a single nanochannel (200 nm × 200 nm cross section) again divided into six 15 µm long sections (Fig. 7a) by extracting the RSID intensity integrated from 510 to 520 nm at the spectral position of the main Fluorescein RSID peak (Fig. 7b) at 22 °C (Fig. 7c), 30 °C (Fig. 7d), 41 °C (Fig. 7e) and 50 °C (Fig. 7f). At the global level, the results are similar for all temperatures, with the channel section closest to the Fluorescein solution microchannel showing the strongest and fastest response, while the RSID intensity from the nanochannels section adjacent to the water-filled microchannel remains essentially unchanged. As the reason, we identify the concentration-gradient-dependent diffusion rate, which causes the formerly water-filled section closest to the Fluorescein-filled microchannel to equilibrate most quickly to the highest concentration of the six monitored nanochannel sections. Accordingly, a linear concentration gradient is eventually established along the nanochannel at all temperatures (Figs. S2, S3), as we also have observed earlier^30^.

Interestingly, however, the time it takes to establish this linear gradient depends on temperature, as expected from the Stokes-Einstein equation that predicts higher diffusion rates of solutes at elevated temperatures^47^. To quantitatively evaluate this effect, we fitted a linear function (red dashed line) to the downwards slope of the RSID time trace of channel section 1 and used the extracted slope as indirect measure for the rate of Fluorescein diffusion (Fig. 7c–f). Subsequently comparing the extracted slopes (Fig. 7g) reveals the expected trend that an increase in temperature and thus molecular mobility, leads to a faster convergence towards the steady state of the diffusion of Fluorescein in the nanochannel.

In summary, this relatively simple experiment demonstrates the ability of our holder to establish active temperature control of the fluidic system on the chip, and the possibility to perform NSS measurements in situ from a single nanofluidic channel with high accuracy, as well as temporal and spatial resolution. This ability, in turn, opens the door to temperature-controlled nanofluidics in combination with optical microscopy that we predict may find application in, e.g., single particle catalysis^41,42^ or studies of protein agglomeration and folding^48,49^.

### Applying an electric field across the fluidic system

**Fig. 8:**
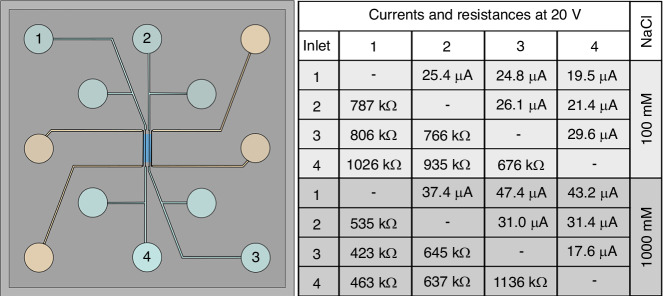
Electric currents through the fluidic system at 20 V for two NaCl concentrations. The ability to insert electrodes into each of the inlet reservoirs enables the use of the fluidic system as an electric system. To demonstrate this, we subsequently filled the sample micro- and nanochannels with 100 mM and 1000 mM NaCl in water solutions, and measured the current between the reservoirs for an applied potential of 20 V. The schematic overview of the fluidic chip indicates the positions of the numbered reservoirs, while the table gives the measured currents and calculated resistances for each possible connection for the two NaCl concentrations. All connections show resistances between 500 kΩ and 1200 kΩ, with the higher NaCl concentration causing lower resistances. The high number of parallel nanochannels creates an effective electric contact between the microchannel connecting reservoir 1 and 4, and the microchannel connecting reservoir 2 and 3. One remarkable exception is the connection between reservoir 3 and 4 for 1000 mM NaCl, where air pockets in the microchannel (Fig. S4) caused an increase in the resistance

As the last part of the experimental assessment of our system, we characterized its ability to generate electric fields across the fluidic system on the chip with the help of macroscopic electrodes that are inserted into selected reservoirs at the corresponding contact points, as depicted in Fig. 3d, e. Here, we used enameled copper wire electrodes. We start out by, in two subsequent experiments, filling the entire experiment fluidic system (i.e., not the reference channel system) with two different concentrations of NaCl in water. In this way, the fluidic channels can be regarded as electric conductors. Subsequently, we measured the electric currents flowing between four selected connection points (Fig. 8) when 20 V are applied to the electrodes in the respective reservoirs (Fig. 8). As expected, the obtained currents increase for the higher molarity NaCl solution. More importantly, however, the currents (and with that the resistances) measured through only microchannels (connection 1–4 and 2–3 in Fig. 8) are very similar to the currents that pass through the nanochannels (e.g., connections 1–2 or 1–3 in Fig. 8). This clearly establishes that the 62 parallel nanochannels in the array function as electric conductors in the same way as the much larger microchannels, since we essentially do not see a significant difference in resistance. This in turn means that it indeed is possible to apply an electric field along (a single) nanochannel(s) by applying a voltage to electrodes located in a liquid-filled reservoir on either side of the nanofluidic system.

On a side note to Fig. 8, we highlight that it is well-established that the electric resistance of a solution is proportional to the concentration of a solute in that solution^50,51^, which means that resistivity measurements can be used to monitor concentration changes inside micro- and nanofluidic systems. Furthermore, for our present application it means that the same principle also can be used to check for fluidic continuity in the channels. As an example, we see that the resistance measured between points 3 and 4 for the 1000 mM NaCl case is about double the average of all the other connections (Fig. 8), especially when compared to the, from a fabrication and length perspective, identical pathway found between points 1 and 2. This hints at an obstruction in the fluidic system, such as air bubbles (see Fig. S4), that only leave a thin liquid layer on the channel walls^30^ to transport the electric current.

Having established the ability of our system to create electric fields along nanofluidic channels, we repeated the diffusion experiment introduced above (cf. Fig. 7) at 50 °C while introducing an electric field. The cathode was placed in the reservoir with the Fluorescein solution, while the anode was inserted in the water-filled reservoir on the other side of the nanochannel array. Since the Fluorescein solution was prepared from Fluorescein disodium salt, the Fluorescein molecules are negatively charged in aqueous solution. This, in turn, is expected to induce a drift of Fluorescein ions towards the cathode, which means that the presence of an electric field is expected to slow down/inhibit the diffusion of Fluorescein into the nanochannels.

**Fig. 9:**
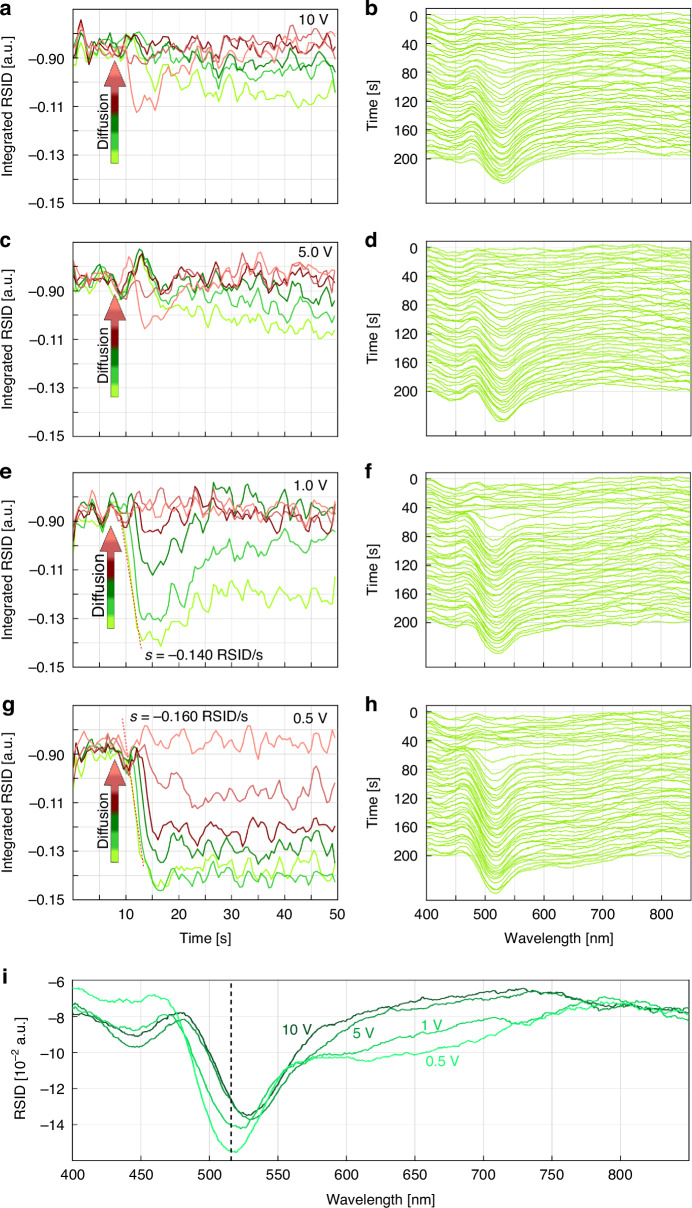
Fluorescein diffusion into water inside a single nanochannel in the presence of an electric field. We used the same nanofluidic channel with 200 nm × 200 nm cross section and the same experimental scheme at 50 °C as in Fig. 8, with two electrodes (coated copper wire, 0.5 mm diameter) inserted into the reservoir of the aqueous Fluorescein solution (reservoir 2 in Fig. 8) and into the water filled reservoir (reservoir 1 in Fig. 8) of the sample nanochannel fluidic system. The cathode (+) is placed in the Fluorescein reservoir while the anode (-) is placed in the water reservoir. Since these two reservoirs are located on either side of the nanofluidic channels on the chip, an electric field can be generated across them, which is oriented such that the (in solution) doubly negatively charged Fluorescein molecules are transported towards the cathode. **a** RSID amplitude integrated between 523 nm and 533 nm, plotted as a function of time for the six color-coded nanochannel sections for an applied potential of 10 V with the corresponding inverted RSID ridge-plot in (**b**). **c**, **d** same as (**a**) but for 5 V applied potential and RSID integration between 527 nm and 537 nm. For 10 V and 5 V the diffusion sets in very slowly after the pressure is released at 10 s, and only reaches a fraction of the RSID at the end of the experiment when compared to Fig. 7f. This is the direct consequence of the electric field counteracting diffusion and thereby preventing Fluorescein molecules from entering the nanochannel. **e**, **f** same as (**a**), but for 1 V and RSID integration between 519 nm and 529 nm. Evidently, diffusion of Fluorescein into the water-filled channel sets in directly at 10 s when the applied pressure is released. However, only channel section 1 closest to the Fluorescein-filled microchannel can maintain a relatively high concentration over time. In all other sections, the Fluorescein concentration decreases again because of the applied field that counteracts diffusion. A linear fit has been applied to the RSID slope of section 1 (red dashed line, Fig. S5). **g**, **h** same as (**a**), but for 0.5 V and RSID integration between 512 nm and 522 nm. Here the observed behavior is very similar to Fig. 7f where no field is applied. Diffusion sets in rapidly at 10 s, when the applied pressures on the system are released, and creates a concentration gradient along the nanochannel. A linear fit has been applied to the RSID slope of section 1 (red dashed line, Fig. S5). **i** Fluorescein RSID spectra measured at 10 V, 5 V, 1 V and 0.5 V applied potential at *t* = 45 s for the nanochannel section closest to the Fluorescein side (section 1). They reveal a distinct spectral red-shift and intensity decrease of the RSID peak for higher potentials that corresponds to the absorption band of the Fluorescein molecule. This is the consequence of field-induced shifts of the energy levels of the conjugated electron system of Fluorescein (as already demonstrated for other molecules with conjugated electron sytems^52–54^). The position of the peak without voltage applied (as shown in Fig. 7b) is indicated with a dashed line

To investigate this scenario experimentally, we again recorded time-traces of the integrated RSID amplitude at the position of the Fluorescein RSID peak (integrated 10 datapoints around the respective peak position) for the six sections of a nanochannel with 200 nm × 200 nm cross section, and for 10 V (Fig. 9a), 5 V (Fig. 9c), 1V (Fig. 9e) and 0.5 V (Fig. 9g) applied potentials. We also plot the corresponding ridge plots of the inverted RSID spectra (Fig. 9b, d, f, h). This reveals that at 10 V applied potential the Fluorescein diffusion is strongly suppressed (Fig. 9a). Only after 30 s (20 s of free diffusion) a small concentration gradient forms along the nanochannel (Figs. S2, S3). The ridge plot (Fig. 9b) of the RSID spectra time evolution from nanochannel section 1 corroborates this result, as the distinct RSID peak for Fluorescein at 520 nm is developing slowly over time (Fig. S3e) and appears at greatly reduced amplitude compared to the free diffusion in absence of electric field (cf. Fig. 7f), indicating a low Fluorescein concentration.

Turning to 5 V applied potential, we find a very similar result (Fig. 9c, d) as this voltage is still high enough to generate an electric field that effectively inhibits the Fluorescein species from entering the nanochannels. Decreasing the potential to 1 V changes the scenario as diffusion now rapidly sets in at 10 s when the pressure is released (Fig. 9e, f), however at a slower diffusion speed than expected for 50 °C. The slope value of the (red dashed) line fitted to the curve of section 1 of −0.140 RSID/s is well below the value of −0.202 RSID/s measured without potential (cf. Fig. 7f, see also Fig. S5). This indicates a retardation, as even the progression of the dye through the nanochannel is halted and eventually reversed, as the RSID intensity time traces decrease in amplitude after 15 s. Only the channel sections 1–3 (green traces) closest to the Fluorescein-filled microchannel maintain a relatively high Fluorescein concentration over time. For channel section 1, we see that a plateau is formed after 30 s, which indicates that a steady state has been reached. The ridge plot for this scenario (Fig. 9f) shows that the RSID spectra are now more distinct and corroborate that diffusion sets in more rapidly.

Finally reducing the applied potential to 0.5 V yields integrated RSID amplitude time traces (Fig. 9g) similar to the ones obtained in the absence of electric field (cf. Fig. 7f), with diffusion rapidly setting in at 10 s and establishing a concentration gradient along the nanochannel. However, the slope value of the fitted line of 0.160 RSID/s (Fig. S5) suggests that the diffusion is still retarded by the electric potential. In addition, this gradient is non-linear (Fig. S2h, Fig. S3h), as the consequence of the weak electric field still preventing completely free diffusion. The ridge plot for this scenario corroborates the rapid diffusion of Fluorescein into the nanochannel at high concentration reflected in the strong RSID peak at 520 nm.

As a final aspect, we note the NSS measurements reveal a spectral red-shift of the main RSID peak attributed to Fluorescein at different applied potentials (Fig. 9i), which means that also the absorption peak (see Fig. S6) is shifted to longer wavelengths. This is an interesting result, since it previously has been shown for other organic molecules that an external electric field can change their molecular electronic structure in such a way that the absorption wavelength is redshifted^52,53^. Hence, our results indicate that the energy gap between the highest occupied molecular orbital and the lowest unoccupied molecular orbital is decreased, such that photons of lower energy (longer wavelength) are required to promote and electron within the molecular system across this gap. We also note that we observe a decrease of the RSID peak amplitude at higher applied potentials. The investigation of other organic molecules in external electric fields^52–54^ suggests that the absorbance tends to increase with an increased external electric field, leading to the conclusion that the RSID decrease is caused by the decreased Fluorescein concentration in the nanochannel at higher potentials and not by a change of the molecule’s electronic structure.

## Conclusions

We have presented a fluidic chip holder that allows the control of temperature, electric field, pressure and liquid flow in an only 1 cm^2^ size fluidic chip with up to 12 fluidic connection points and compatibility with in situ optical microscopy and spectroscopy readout. Specifically, we have demonstrated how Peltier-elements can be used to vary the temperature of the fluidic chip between 12 °C and 112 °C, how electric fields can be introduced into the fluidic micro- and nanochannels by connecting electrodes to fluidic connection points, and how NSS can be used for time-resolved in situ optical readout of the spectral fingerprint of a liquid inside a single nanochannel on the chip. To showcase these functions in detail, we used NSS to record scattering spectra from up to six sections of a single nanochannel and employed Brilliant Blue and Fluorescein as model species. Using them, we demonstrated the (i) NSS monitoring of on-chip mixing and exchange of a dye solution; (ii) temperature-dependence of Fluorescein diffusion into a single water-filled nanochannel, and (iii) Fluorescein diffusion into water inside a single nanochannel in the presence of an electric field. As key results, we corroborated higher Fluorescein diffusion rates at higher temperatures, and that applied electric fields along a nanochannel effectively inhibit the diffusion of a charged molecule like Fluorescein in solution into a single nanochannel.

From the more general perspective of micro and nanofluidics, we demonstrated that a multifunctional fluidic chip holder in combination with a highly customizable, while at the same time only 1 cm^2^ size, fluidic chip offers a multitude of experimental options in combination with scalable micro- and nanofabrication. The range of possible future applications of the here presented chip and chip holder is wide, as it is versatile and adaptable to specific experimental designs. We first and foremost envision this chip holder to be used for heterogenous catalysis on single nanoparticles in model pores^27^, perhaps in combination with microscale electrochemistry^55^. Notably, however, on-chip mixing and readout methods such as NSS enable investigations within homogenous catalysis^19^ as well, especially when reaction rates at different temperatures are of interest. In addition, many of the topics within biology that employ micro- or nanofluidics can be investigated with this chip holder and adapted fluidic chip designs, for example protein dynamics^16,17^ and enzymatic activity^18^. Even more physics-based research, such as nanoscale diffusion of multicomponent^56,57^ or asymmetric^58^ electrolytes at different temperatures or electric potentials can be conducted, including electrophoresis or electroosmosis^9–11^ and microfluidic pumps^14^, possibly in connection with fluidic field-effect transistors^15^ or valves on the nanoscale^20^. We predict that multifunctional and integrated fluidic platforms of the type reported will be key enablers for further advances in the micro- and nanofluidics domain both in science and in emerging technical applications.

## Methods

### Instruments

The temperature measurements for the evaluation of the heating cooling system were performed with a METEX M-3850 and a CHY 21C multimeter using a K-type thermocouple. As heat conduction paste, we used ARCTIC MX-2. Electric currents and voltages were measured with a MASTECH MAS830L and an AMPROBE 5XP-A multimeter. For the temperature performance tests, the chip holder was mounted on a MCL RM21 inverted microscope. The NSS measurements were taken on a Nikon Eclipse LV150N upright microscope with a Nikon 50x ELWD dark-field objective. The light source was a Thorlabs Solis-3C LED lamp with a output power of 4 W. The scattered light from the nanochannels was spectrally resolved in an Andor grating spectrometer (SR-193I-A-SL) with a 150 l/mm grating and subsequently recorded with a Andor Newton (DU920P-BEX2-DD) camera attached to the spectrometer. The center wavelength of the spectrometer was set to 600 nm and the exposure time for each frame was 2 s, respectively 0.5 s for the diffusion measurements. Using the multitrack-feature of the camera, the image was divided into 8 tracks, 1 for each reference side and 6 for the sample nanochannel. Each track (21 pixels corresponding to 15 µm of nanochannel) was binned to give one spectrum per track. Pressure to the reservoirs was applied with a Fluigent MFCS-EX pressure controller using nitrogen gas. The injection of water and dye solution was realized with B.Braun Sterican Hypodermic 21 G × 4.75” needles and syringes. Absorbance spectra of the dyes were recorded on a Varian Cary 500 spectrophotometer.

### Preparation of Brilliant Blue and Fluorescein solutions

The Brilliant Blue and Fluorescein dyes were bought as their sodium salts from Merck and diluted into stock solutions of 50 mM concentration with ultrapure water (Milli-Q IQ 7000 water purification, Merck).

### Fluidic chip fabrication

The chips with the micro and nanofluidic systems used in the experiments were fabricated in the clean room facilities of MC2 at Chalmers. 52 fluidic chips could be produced from one 4-inch silicon wafer. The wafer was prepared with a thermal oxide layer into which the fluidic structures were later etched. The fabrication procedure of similar fluidic chips is described in more detail in our earlier work by Levin et al.^26^. Short summary: The 1 mm thick 4-inch (100) silicon wafer was cleaned with Standard Clean 1, followed by a 2% HF dip and Standard Clean 2. A thermal oxide layer of 275 nm thickness was grown at 1050 °C in wet atmosphere. The nanochannels were first patterned in a resist layer with electron beam lithography, transferred into an underlying 20 nm thick Cr hard mask layer with chlorine-based reactive ion etching (RIE), and then transferred into the thermal oxide layer with fluorine-based RIE. For the microchannels, photoresist was patterned with direct laser lithography, and the pattern was transferred into the substrate with RIE. The inlet holes were defined with direct laser lithography into a photoresist layer and etched through the wafer with deep reactive ion etching. Finally, the substrate and a 175 µm thick Borofloat 33 glass wafer were treated with Standard Clean 1, with the glass wafer being the lid for the fluidic system. Both wafers were then treated with O_2_ plasma (1 min, 50 W RF power, 250 mTorr) to facilitate the pre-bonding of the glass cover lid to the wafer with the fluidics. The final fusion bonding was carried out at 550 °C for 5 h. After bonding the fluidic wafer was cut into 52 quadratic chips to be used in the chip holder.

## Supplementary information

Supplementary information for publication

## Data Availability

The underlying data for this publication is available at Zenondo, 10.5281/zenodo.12204566.
