# Supplementary material for: A temperature-controlled chip holder with integrated electrodes for nanofluidic scattering spectroscopy on highly integrated nanofluidic systems
Source: Microsyst Nanoeng. 2026 Jan 19;12:32. doi: 10.1038/s41378-025-01125-9 (PMC12816118; doi:10.1038/s41378-025-01125-9)
Supplement: Supplementary file 1 — Supplementary information for publication [file 41378_2025_1125_MOESM1_ESM.docx]

**Supplementary Material for**

A temperature-controlled chip holder with integrated electrodes for nanofluidic scattering spectroscopy on highly integrated nanofluidic systems

*Björn Altenburger^1^, Joachim Fritzsche^1^ and Christoph Langhammer^1*^*

^1^Department of Physics, Chalmers University of Technology; SE-412 96 Gothenburg, Sweden

*Corresponding author: clangham@chalmers.se


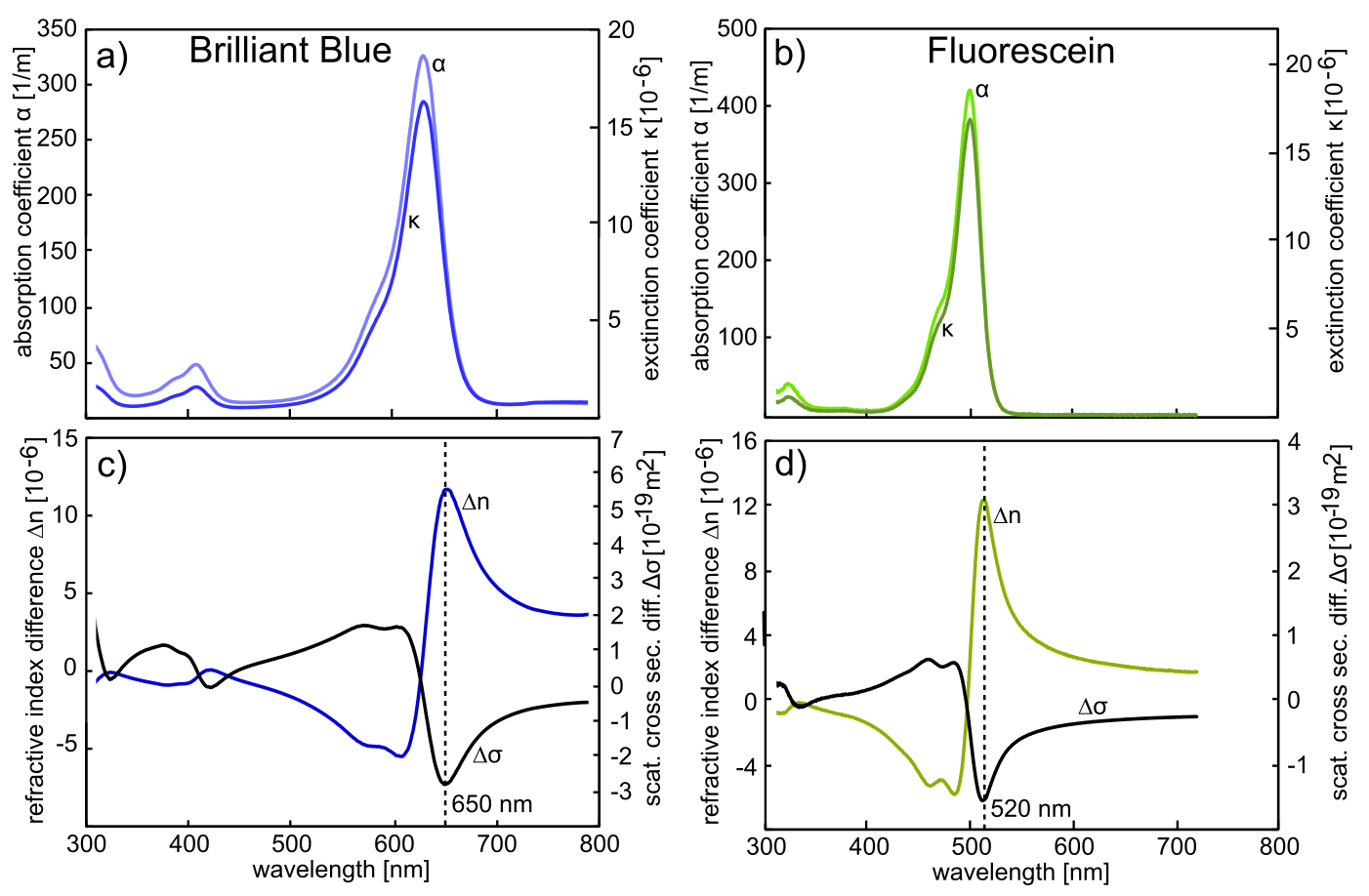


**Figure S1. *Absorption coefficient, extinction coefficient, refractive index change and nanochannel scattering cross section change as function of wavelength for Brilliant Blue and Fluorescein.*** *a) Absorption and extinction coefficient spectrum of Brilliant Blue (11 µM aqueous solution). b) Absorption and extinction coefficient spectrum of Fluorescein (24 µM aqueous solution). c) Calculated difference in the real part of the RI spectrum compared to water for Brilliant Blue together with the corresponding difference of the scattering cross section of a nanochannel. d) Calculated difference in the real part of the refractive index spectrum compared to water for Fluorescein together with the corresponding difference of the scattering cross section of a nanochannel.*

The absorbance spectra in **Figure S1a,b** were measured using a Varian Cary 50 Bio instrument and are here depicted as absorption coefficient, α(*λ*), spectra. α(*λ*) can be calculated by dividing the measured absorbance values by the optical path through the sample solution (here 1 cm) and log(e). The subsequent calculation of the extinction coefficient, κ (*λ*), is done as

|  | $\kappa\left( \lambda\right)=\frac{\alpha(\lambda) \lambda}{4\pi}$ | *Equation S1* |
| --- | --- | --- |

Subsequently using κ (*λ*) in the Kramers-Kronig relation

|  | $\Delta n\left( \lambda\right)=n\left( \lambda\right)-1=\frac{2}{\pi}\mathcal{P}\int_{0}^{-\infty} \frac{\kappa\left( \lambda´ \right)}{\lambda´\left( 1-\left( \frac{\lambda´}{\lambda} \right)^{2} \right)}d\lambda´$ | *Equation S2* |
| --- | --- | --- |

yields the change of the real part of the refractive index caused by light absorption by Brilliant Blue (**Figure S1c**) or Fluorescein (**Figure S1d**) molecules in the solution. This change in the real part of the refractive index is subsequently used in Equation S3, that describes the scattering cross section of a nanochannel, $\sigma_{\mathrm{channel}}$, in relation to the RI difference between the channel and its surrounding^1^. Here, $m={n_{\mathrm{sol}}}/{n_{SiO2}}$ is the ratio of the RIs of the solution in the channel, $n_{\mathrm{sol}}$, and of the surrounding medium of the channel, which is SiO_2_ in the present case, $n_{SiO2}$. The RI of the solution is the sum of the RI of the solvent, H_2_O, and the calculated $\Delta n$ from above, $n_{\mathrm{sol}}= n_{H2O}+\Delta n$.

|  | $\sigma_{channel,solution}=\frac{A_{\emptyset}^{2}k^{3}L}{4}\left( m^{2}-1 \right)^{2}\left( \frac{1}{2}+\frac{1}{\left( m^{2}+1 \right)^{2}} \right)$ | *Equation S3* |
| --- | --- | --- |

Furthermore, $\sigma_{\mathrm{channel}}$ is dependent on the geometrical channel cross section, $A_{\emptyset}$, its (illuminated) length, $L$, and the wavenumber of the incident light, $k=2\pi/\lambda$.

To finally calculate only the contribution from the dye absorption to the scattering cross section of the nanochannel, Equation S3 is employed again. However, now only the RI of water is used $n_{\mathrm{sol}}= n_{H2O}$ to yield $\sigma_{channel,water}$. Subtracting then from $\sigma_{channel,solution}$ the scattering cross sectionof the only water-filled channel yields the difference in scattering cross section, $\Delta\sigma$ = $\sigma_{channel,solution}-\sigma_{channel,water},$ which is caused by the dye absorption. Since the experimentally measured difference in scattering intensity (RSID) is proportional to $\Delta\sigma$ (as $I_{scat}\sim\sigma_{scat}$), the calculated $\Delta\sigma$ spectra can be compared to the RSID spectra. In **Figure S1c,d**, the position of the main $\Delta\sigma$ peak is indicated with a dashed line and is used in the main text to identify the two different dyes.


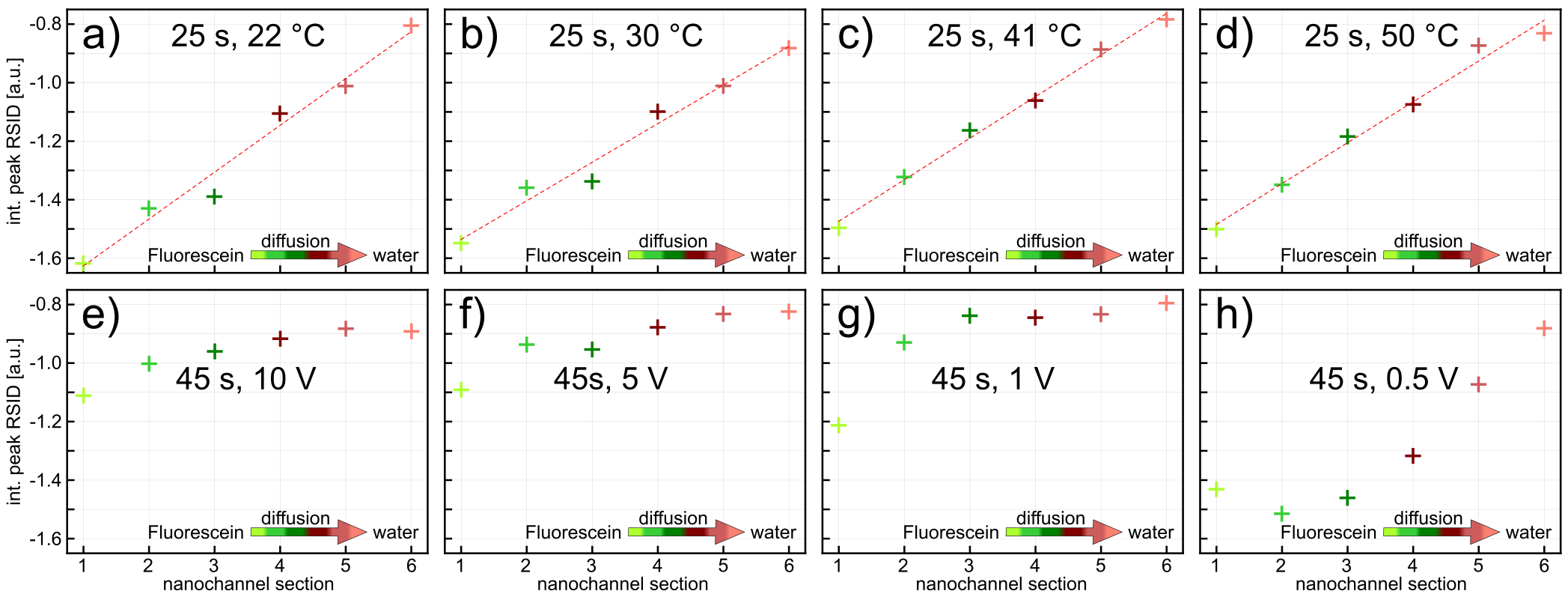


**Figure S2. *Integrated peak RSID values for the six nanochannels sections of Figures 7 and 9.*** *a-d) Integrated RSID peak values for the diffusion measurements at different temperatures as presented in* ***Figure 7*** *in the main text. The values shown here are taken at 25 s (15 s after diffusion start) and display a linear correlation, as demonstrated by the line fit (red dashed line), which means that the final steady state of the diffusion along a nanochannel has been reached. e-h) Same as for a-d, but for the diffusion measurements at 52 °C with different applied voltages as shown in the main text* ***Figure 9*** *and taken at 45s (35 s after diffusion start). The significant difference between the examples shown in a)-d) and e)-f) become apparent, as the data points are now arranged at a much lower angle, if a linear correlation is still assumed. The reason for this is the influence of the electric field, as discussed in the main text, which inhibits the diffusion of the charged dye species into the nanochannel, such that the RSID amplitude is much smaller when compared to the diffusion without electric field. In g), when the voltage at the inlet reservoirs is reduced to 1 V, the integrated RSID value for the first nanochannel sections (closest to the dye-filled microchannel) is higher than for 10 V and 5 V, showing that the Fluorescein molecules do enter the nanochannel but still the diffusion is suppressed significantly by the electric field. In h), when the voltage is reduced to 0.5 V, the dye species reach all sections of the nanochannel and a linear correlation can be seen again, but only for sections 3 and onwards. As the integrated RSID values suggest, the Fluorescein concentration in the first three sections of the nanochannel is similar and high, indicating that the diffusion can readily proceed until a certain point along the nanochannel, which also means until a certain electric field strength, as there is a voltage drop across the nanochannel.*

*
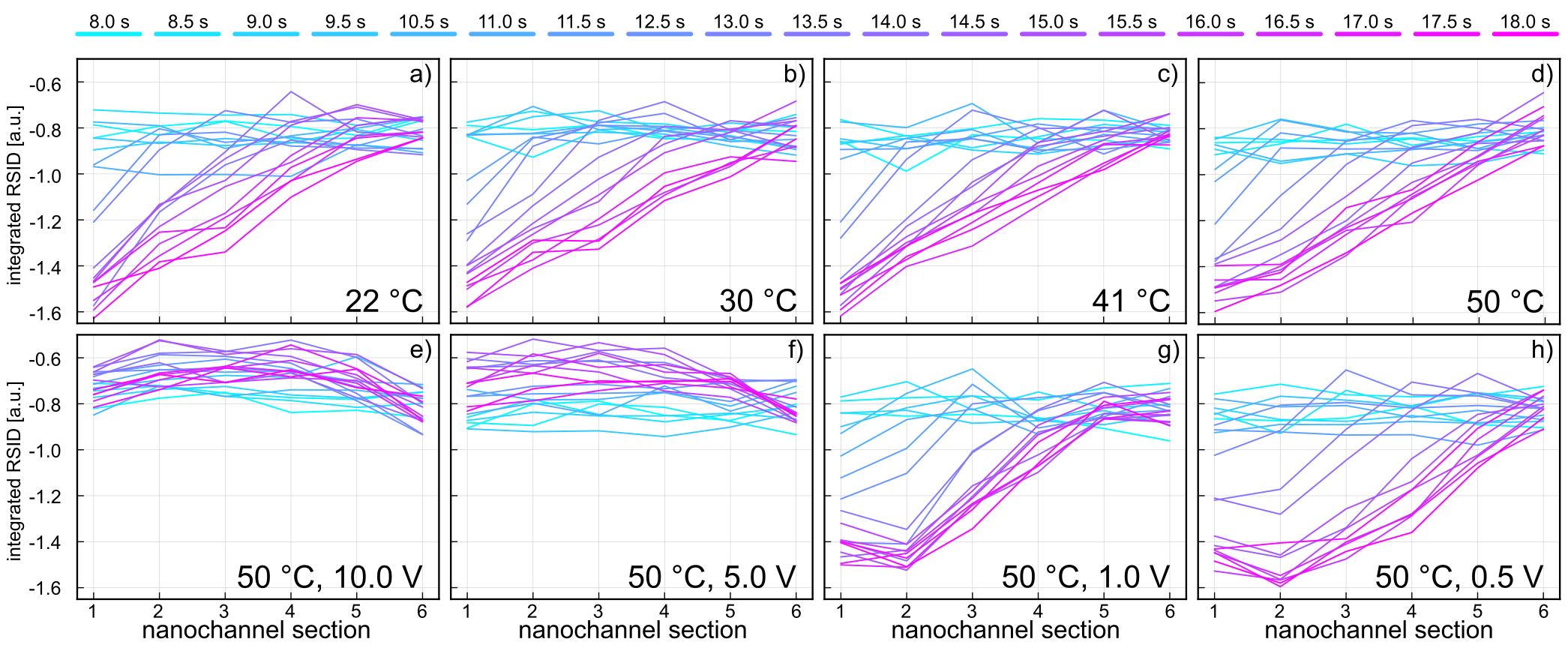
*

**Figure S3. *RSID gradient along the nanochannel over time.*** *a-d) Integrated RSID peak values for the diffusion measurements at different temperatures as presented in* ***Figure 7*** *in the main text. Here, each line represents the integrated RSID for the six nanochannel sections for a specific time between t = 8.0 s and t = 18.0 s, with the diffusion start at t = 10 s. The progression for the different temperatures is similar, whereas the steady state is reached slightly faster for higher temperatures, as discussed for* ***Figure 7****. e-f) Same as for a-d, but with the temperature kept at 50 °C and electric potential applied as detailed in* ***Figure 9****. Here, the diffusion of Fluorescein into the nanochannel is prevented for 10 V and 5 V, since the RSID along the nanochannel remains constant over time. For 1 V, the diffusion sets in, but does not reach a steady state, as shown in a)-d). For 0.5 V, the result is similar, but Fluorescein seems to be able to enter deeper into the nanochannel. However, a linear gradient along the nanochannels is not reached.*

*
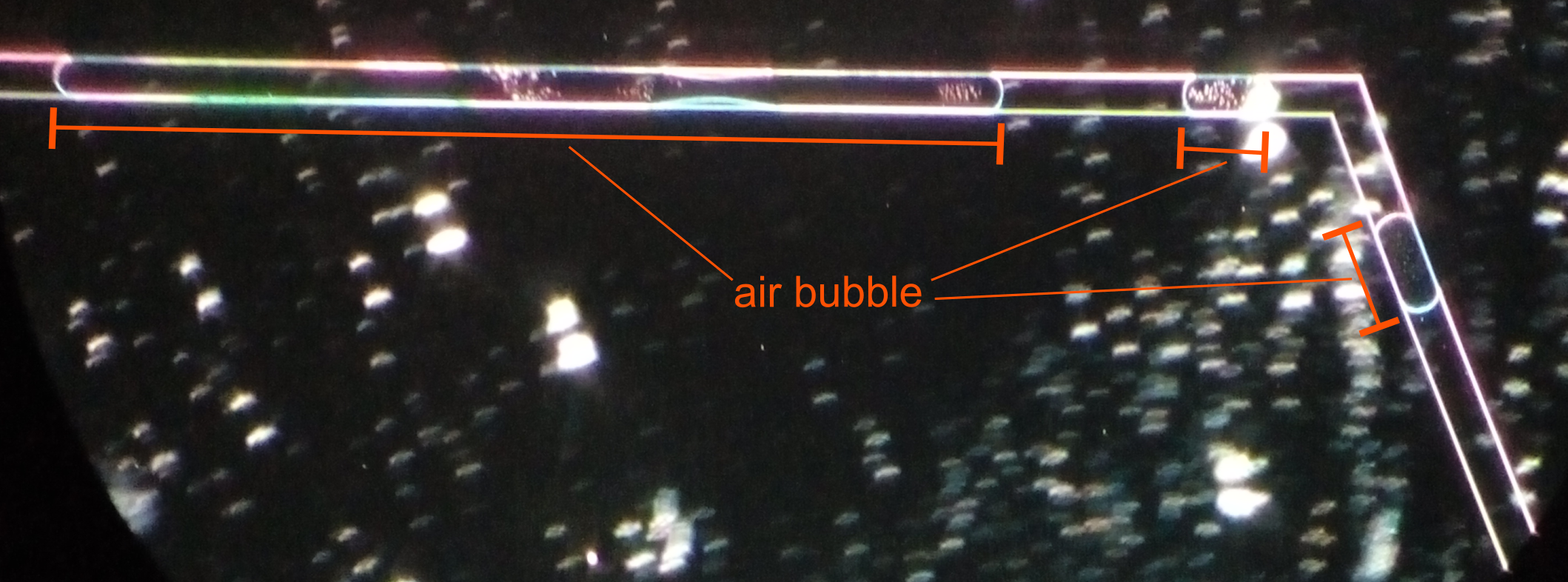
*

**Figure S4. *Microchannel being partly obstructed by air bubbles*.** *This darkfield image is taken from the microchannel leading to reservoir 1 (see* ***Figure 8*** *in the main text) after the measurement with 1000 mM NaCl in water was concluded. The obvious presence of several air bubbles corroborates them being the reason for the measured low current.*


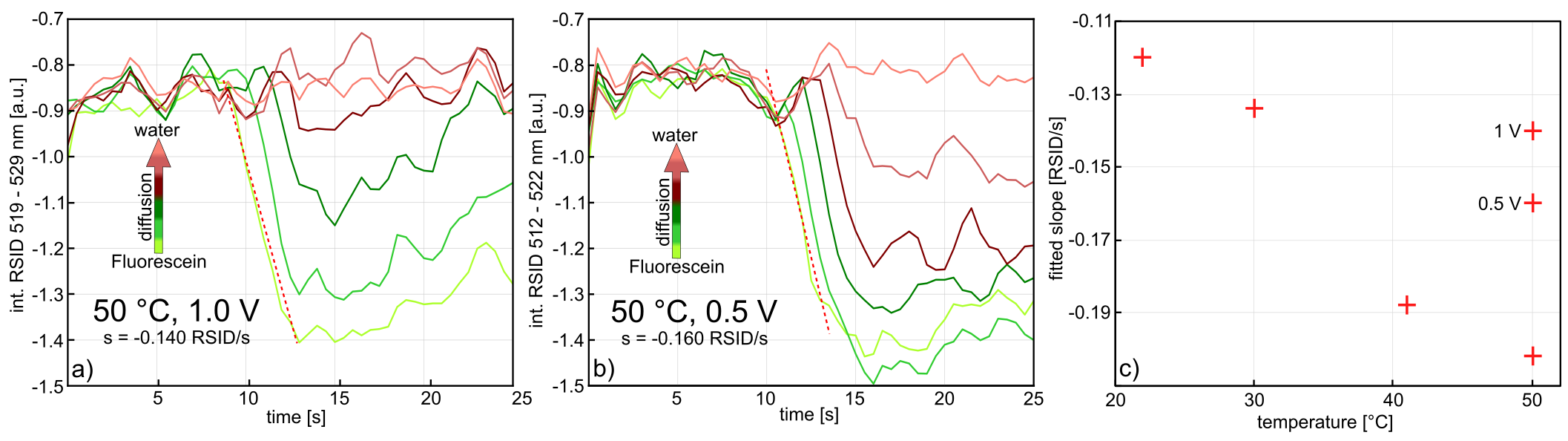


**Figure S5. *Supplementary figures for Figure 9.*** *a) RSID amplitude integrated between 519 nm and 529 nm, plotted as a function of time for the six color-coded nanochannel sections for an applied potential of 1.0 V, zoomed in for a linear fit of the diffusion slope of section 1. b) Same as a), but for the diffusion measurement at 0.5 V. c) Comparison of the slope values (RSID change per unit time) for the different temperatures as shown in* ***Figure 7g****, but here with the slope values of a) and b) added for comparison. The applied potential causes a retardation of the diffusion, since the values for 1 V and 0.5 V are well below the value previously measured for 50 °C with no potential applied.*

***
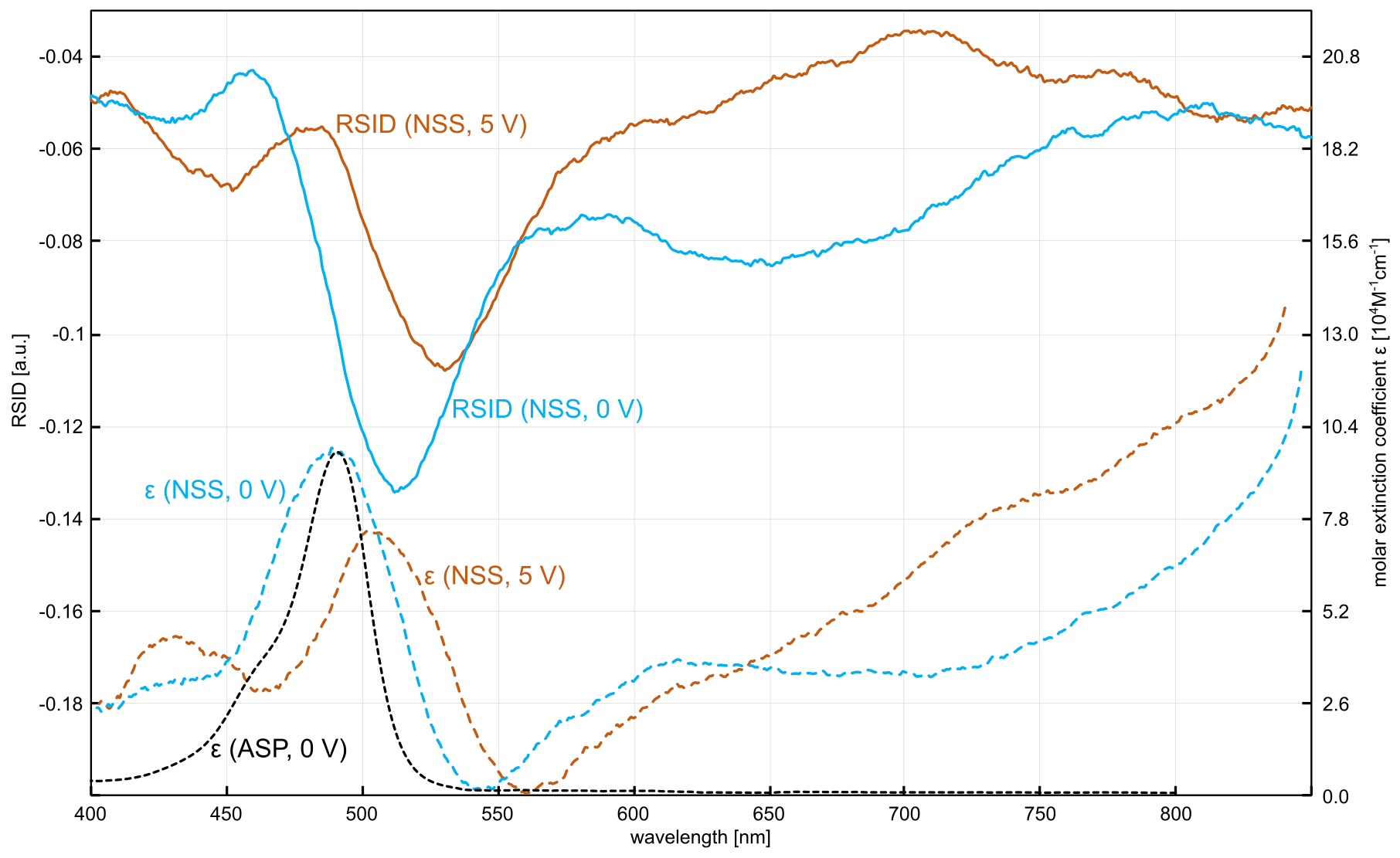
***

**Figure S6. *Comparison of RSID and molar extinction coefficient spectra of Fluorescein for different applied electric potentials.*** *RSID spectra of Fluorescein taken at t = 45s (35 s after diffusion start, see* ***Figure 9i****) and applied potential of 0 V and 5 V respectively. The corresponding molecular extinction coefficient (ε) spectra have been calculated from the RSID spectra according to the process given in our previous work*^1^ *and are shown here together with the ε spectra from ASP. When no voltage is applied across the nanochannel, the peak positions of the ε spectra from ASP and NSS match well. At an applied voltage of 5V, the ε peak appears shifted towards longer wavelengths by 15 nm, indicating a significant change in the electronic structure of the molecule as discussed in the main text.*

Supplementary References

1. Altenburger, B., Fritzsche, J. & Langhammer, C. Visible Light Spectroscopy of Liquid Solutes from Femto- to Attoliter Volumes Inside a Single Nanofluidic Channel. *ACS Nano* **19**, 2857–2869 (2025).
